# Biomarker testing in lung cancer: from bench to bedside

**DOI:** 10.3389/or.2024.1445826

**Published:** 2025-01-06

**Authors:** Ullas Batra, Shrinidhi Nathany

**Affiliations:** ^1^ Medical Oncology, Rajiv Gandhi Cancer Institute and Research Centre, New Delhi, India; ^2^ Hematology and Bone Marrow Transplant, Fortis Memorial Research Institute, Gurgaon, Haryana, India

**Keywords:** biomarker, next-generation sequencing, targeted therapy, lung cancer, precision oncology

## Abstract

Non-small-cell lung cancer (NSCLC) is the poster child of personalized medicine. With increased knowledge about biomarkers and the consequent improvement in survival rates, NSCLC has changed from being a therapeutic nihilistic disease to that characterized by therapeutic enthusiasm. The routine biomarkers tested in NSCLC are EGFR, ALK, and ROS1. However, several additional biomarkers have been added to the diagnostic landscape. Current guidelines recommend testing at least seven biomarkers upfront at the time of NSCLC diagnosis—emphasizing the wide range of targets and corresponding therapies that can be leveraged for disease management. Sequential single-gene testing is not only time-consuming but also leads to tissue exhaustion. Multigene panel testing using next-generation sequencing (NGS) offers an attractive diagnostic substitute that aligns with the evolving dynamics of precision medicine. NGS enables the identification of point mutations, insertions, deletions, copy number alterations, fusion genes, and microsatellite instability information needed to guide the potential use of targeted therapy. This article reviews the existing guidelines, proposed recommendations for NGS in non-squamous NSCLC, real-world data on its use, and the advantages of adopting broader panel-based NGS testing over single-gene testing.

## Introduction

At the beginning of this century, the survival rate of a stage 4 lung cancer patient was a dismal 1 year (1). However, with increasing knowledge about the biomarkers in lung cancer, there has been a paradigm shift in the prognosis and survival rates of these patients.

Currently, polymerase chain reaction (PCR)-based methods and conventional direct sequencing methods like Sanger sequencing and pyrosequencing are employed to identify these biomarkers. However, these approaches allow sequencing of a few genes per run, resulting in a technically cumbersome, time-consuming, and expensive diagnostic test. Multigene sequencing using next-generation sequencing (NGS), also known as massively parallel sequencing, avoids performing multiple sequential single tests for all these biomarkers. It has advantages such as sparing tissue samples, avoiding delays for patients, and helping match the patient to the most appropriate clinical trial. Although cost, slow turnaround time, and the enormity of data returned are certain issues, the pieces of information and advantages outweigh the same.

This article reviews the concepts of precision oncology in non-small-cell lung cancer (NSCLC), with an in-depth description of canonical biomarkers, advantages and disadvantages of NGS-based testing for the biomarkers, and current recommendations.

## Understanding NGS

**FIGURE 1:**
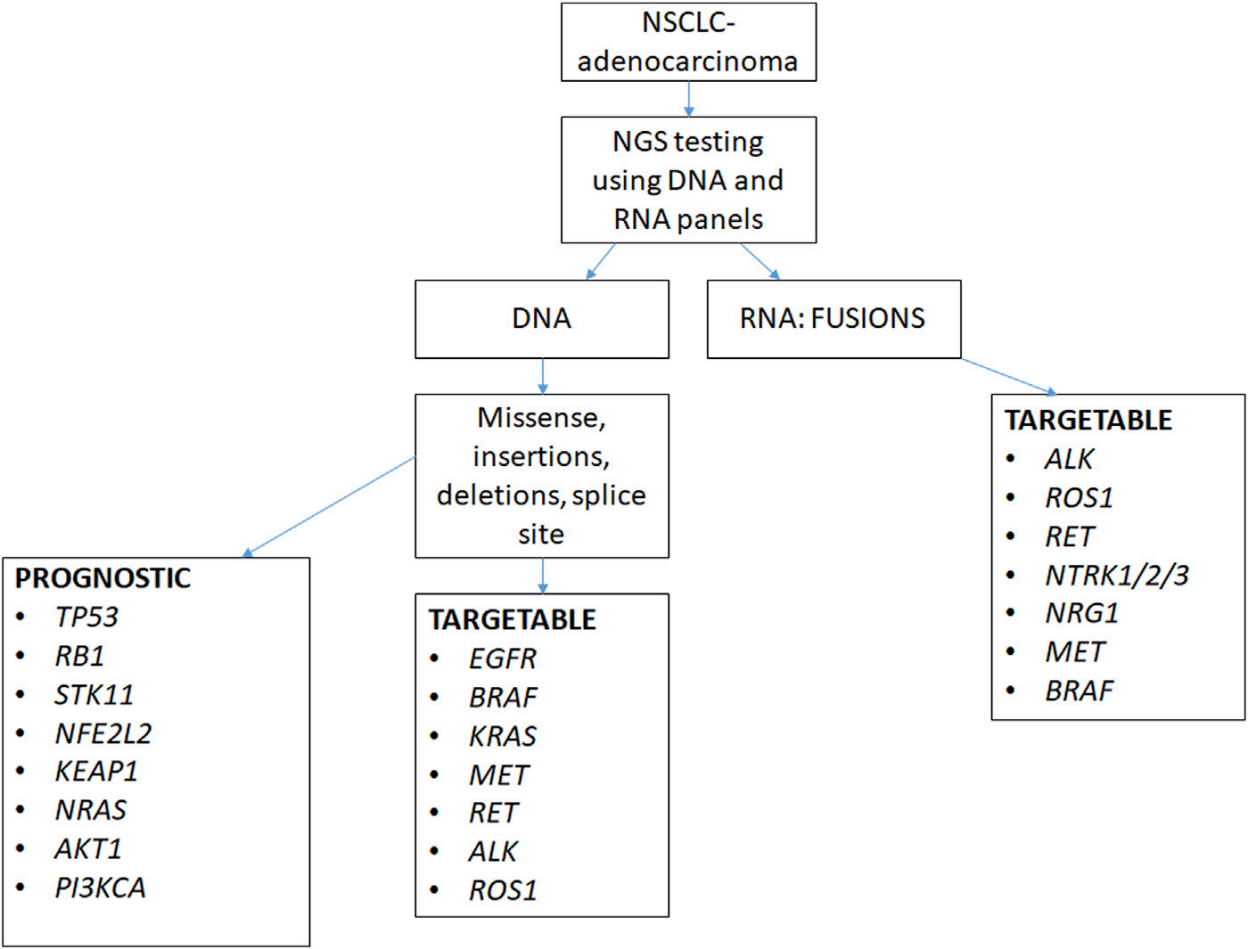
Flow diagram depicting the steps involved in NGS. Step 1: extraction, Step 2: library preparation, Step 3: bioinformatics, and Step 4: final clinical report. *DNA, deoxyribonucleic acid; RNA, ribonucleic acid; PCR, polymerase chain reaction; HGVS, Human Genome Variation Society; ACMG/AMP, American College of Medical Genetics and Genomics/Association of Molecular Pathology; VUS, variant of uncertain significance*.

The NGS process involves three main phases (2) (Figure 1):

1. *Sample preparation*: DNA/RNA extraction, target region capture/fragmentation, and library preparation.
2. *Sequencing process*: Each library fragment is read multiple times from either one end (single end) or from both ends (paired end).
3. *Bioinformatics*: This is the most critical phase. This involves the alignment of the reads to the reference genome using specific algorithms; filtering low-quality data; coverage, which reports the number of reads for each region that is sequenced; variant calling, which reports all the genetic variants using specific software applications like GATK best practices; and, finally, the annotation, which defines and links the variants to the disease in context.

## Current recommendations for NGS testing in NSCLC

The 2018 CAP/AMP/IASLC (College of American Pathologists/Association of Molecular Pathology/International Association for the Study of Lung Cancer) guidelines (3) advocated testing for *EGFR*, *ALK*, *ROS1*, and *BRAF* upfront. Currently, NGS is offered only when sequential single-gene testing yields negative results. However, with the advent of newer drugs, the Spanish Society of Medical Oncology (4) has advocated for upfront panel-based testing. ACMG/AMP (5, 6) (American College of Medical Genetics and Genomics) guidelines are employed to ascertain the pathogenicity of the called variant and are detailed in Table 1.

**TABLE 1:** Classification of somatic variants in cancer based on pathogenicity as per ACMG/AMP guidelines and FDA approval of drugs.

Tier	Category	Evidence
Tier I	Variant of strong clinical significance	Level A: FDA-approved therapy, included in professional guidelines Level B: Well-powered studies with consensus from experts in the field
Tier II	Variant of potential clinical significance	Level C: FDA-approved therapy for different tumor types or investigational therapies Level D: Pre-clinical trials or a few case reports with consensus
Tier III	Variant of unknown clinical significance	Not observed at a significant allele frequency in the general population or subpopulation databases, or pan-cancer or tumor-specific variant databases No convincing published evidence of cancer association
Tier IV	Likely benign/benign	Observed at a significant allele frequency in the general population or subpopulation databases No existing published evidence of cancer association

ACMG/AMP, American College of Medical Genetics and Genomics/Association of Molecular Pathology; FDA, Food and Drug Administration.

## Biomarkers in NSCLC

Presently, clinical biomarker testing in NSCLC involves investigating any potential druggable alterations (7). The comprehensive genomic profiling of DNA and RNA using NGS panels allows almost complete detection of all these alterations. The various biomarkers, their corresponding drugs, and testing modalities are listed in Table 2.

**TABLE 2:** Biomarkers in non-small cell lung cancer with corresponding targeted drugs and testing modalities.

Biomarker	Drug	Testing modality
*EGFR*	Gefitinib, erlotinib, afatinib, and osimertinib	Real-time PCR—therascreen and Roche cobas v2 Oncomine Target Test FoundationOne Dx
*ALK*	Crizotinib, ceritinib, alectinib, brigatinib, and lorlatinib	IHC, FISH, RT-PCR, and NGS
*ROS1*	Crizotinib, lorlatinib, repotrectinib, and entrectinib	IHC, FISH, RT-PCR, and NGS
*MET*	Crizotinib, capmatinib, savolitinib, and tepotinib	FISH and NGS
*BRAF*	Dabrafenib and trametinib	Real-time PCR, IHC, and NGS
*ERBB2*	Trastuzumab, poziotinib, and tarloxotinib	NGS
*KRAS*	Sotorasib and adagrasib	NGS
*NTRK*	Entrectinib and larotrectinib	IHC, FISH, and NGS
*RET*	Selpercatinib	NGS

IHC, immunohistochemistry; FISH, fluorescence in situ hybridization; RT-PCR, real-time polymerase chain reaction; NGS, next-generation sequencing.

## DNA-based alterations: mutations (single-nucleotide variations, insertions, and deletions)

### EGFR

Epidermal growth factor receptor (*EGFR*)-mutated NSCLC is a distinct molecularly refined subgroup, first described in 2004 (8). These mutations are known to occur in 33%–35% of Asians (9) and 8%–10% in the West (9). Affected patients are usually female individuals, non-smokers, and have adenocarcinoma histology, although the absence of these characteristics should not preclude testing for *EGFR* mutations. Almost all *EGFR* mutations span across exons 18–21 (10). Approximately 90% of the activating mutations detected in *EGFR* are p. L858R and in-frame deletions in exon 19 of the gene (11). The other mutations detected in exons 18, 20, and 21 of the gene are grouped as “uncommon mutations,” some of which are sensitive to tyrosine kinase inhibitors (TKIs), whereas exon 20 insertions are not sensitive to the abovementioned TKIs (12). Drugs like amivantamab are being used for these mutations (13).

Detection methods include real-time PCR-based testing of tumor tissue using FDA-approved CDx like therascreen and Roche cobas v2 tests. However, these are limited by the spectrum of mutations covered by the primer–probe sets, and hence, any complex indels and other rare mutations may be missed. Sanger-based sequencing, although still considered the gold standard, is limited by its sensitivity and user-dependent variability. In our experience, approximately 10% of cases are missed with single-gene testing, which were subsequently detected with broader panel-based NGS testing.

The sensitizing mutations can be targeted using EGFR TKIs like gefitinib, erlotinib, afatinib, and osimertinib. Many randomized controlled trials have demonstrated the superior efficacy and safety of these over platinum-based chemotherapy. Phase III clinical trials, such as IPASS (14), WJTOG (15), NEJ002 (16), EURTAC (17), LUX-Lung3 (18), and LUX-Lung6 (18, 19), have demonstrated a median progression-free survival (PFS) rate of 9–13 months and overall survival times that exceed 24–30 months. Despite all the advantages, resistance mutation p. T790M (exon 20) develops in these patients, which can be targeted using osimertinib (a third-generation TKI). This was investigated in AURA 3 (20) and demonstrated a benefit over pemetrexed and platinum-based therapy with a median PFS rate of approximately 10.4 months in the osimertinib subgroup.

With the results of the FLAURA trial (21), osimertinib (a third-generation TKI) treatment, in the first line, has been shown to have a longer PFS than other comparator *EGFR* TKIs (median duration, 18.9 months vs 10.2 months; hazard ratio for disease progression or death, 0.46; *P*< 0.001) with a better safety profile and overall survival (OS) of 38.1 months vs 32 months in the comparator arm. Resistance to osimertinib in the form of p. C797S mutation has also been described (22). Other resistance mutations rarely described include p. L692V, p. E709K, p. L718E/V, p. L792F/H/V, p.G796D/S/R, p. C797G, and p. L798I (22). Apart from these *EGFR-*dependent mechanisms, *EGFR*-independent mechanisms, including *MET*, *PIK3CA*, *BRAF*, and *KRAS* mutations, can also cause resistance (23). With all this evidence, NGS testing becomes relevant in order to detect any potential resistance mechanisms upfront and avoid over/under treatment.

### BRAF

Activating mutations in *BRAF* have been reported in 2%–8% NSCLC cases (24), with almost 50% of them being p. V600E mutations in exon 15 of the gene. Other activating mutations include p. G469X, p. L597R, and p. K601E, as well as impaired mutations like p. G466V, p. D594X, and p. G596C, which are found across exons 11–15 of the gene (25). The single-gene PCR-based technique is currently approved as a companion diagnostic for the treatment of melanoma. The emergence of BRAF mutation has also been reported as a resistance mechanism to other targeted TKIs like *EGFR* TKIs in NSCLC. In NSCLC, two platforms, namely, the Oncomine Dx Target Test and FoundationOne Liquid, are approved for the initiation of targeted therapy.

Targeted therapy in the form of dabrafenib and trametinib in NSCLC is currently recommended only for p. V600E mutations and not for the other rare variants (26). The ESMO recommendations advocate dabrafenib combined with trametinib for *BRAF*-inhibitor-naive patients with *BRAF* p. V600-mutated NSCLC (stage IV) (27). A trial investigating encorafenib and binimetinib in *BRAF* p. V600E-mutated NSCLC is still under study (NCT3915951).

### MET

*MET* is a receptor tyrosine kinase, and its dysregulation may involve gene amplifications, MET exon 14 splice site alterations, *MET* exon 14 skipping mutations, and missense variants in the TK domain (28). *MET* exon 14 alterations are detected in 3%–4% of cases of NSCLC, and amplifications are found in approximately 1%–5% of cases (28). Cases of pulmonary sarcomatoid carcinoma almost have recurrent *MET* exon 14 alterations. *MET* alterations can be both primary drivers of the oncogenic process and secondary resistance mechanisms to EGFR TKIs in EGFR-mutated NSCLC. Hence, testing for *MET* in both these situations is quintessential.

Currently, recommended testing for amplification includes FISH. For exon 14 skipping mutation, capmatinib is the only FDA-approved drug, and FoundationOne CDx is the only approved companion diagnostic for the same. Additionally, direct Sanger sequencing may also be used to detect tyrosine kinase domain alterations. However, various targeted NGS panels, including DNA and RNA, can easily detect *MET* exon 14 alterations and, hence, can be employed.

Clinical trials studying the efficacy of *MET* TKIs in the treatment of patients with *MET* exon 14 mutant NSCLC include studies of crizotinib (NCT00585195), capmatinib (Geometry Mono 1 (29)) (NCT02414139), tepotinib (NCT02864992), and savolitinib (NCT02897479). They have shown a response rate to type I TKIs ranging from 32% to 68%. Early results show a median (PFS) ranging from 5.4 months to 12.2 months depending on the drug and the line of therapy.

### KRAS

Gain-of-function mutations in the *KRAS* gene encompassing exons 2–4 occur in almost 30% of cases of NSCLC (10), alone or in combination with other drivers. These patients are usually female individuals and young; however, no race/histology-specific associations have been described. Transversion p. G12C and p. G12V mutations are known to occur in smokers, whereas transition p. G12D mutations are known to occur in non-smokers (30). Concurrent *TP53* and *STK11* alterations are known to occur with a high mutation burden in smokers (31).

However, recently, the directly targeting mutant *KRAS* has been studied, and new drugs targeting *KRAS* p. G12C are in the pipeline for approval. G12C occurs in 14% of cases of lung adenocarcinoma, and the KRYSTAL-1 (NCT03785249) phase I/II trial tested the agent (32) adagrasib (MRTX849). Another drug sotorasib (AMG-10) has been tested in the CodeBreak 100 trial (33) (NCT03600883) with an ORR of 32.2%. The FDA has approved sotorasib as a new drug after testing for p. G12C using an FDA-approved test, following at least 1 prior line of systemic therapy. Testing for this mutation, hence, is clinically relevant, and DNA-based NGS panels incorporate it.

### ERBB2 (Her2/neu)

*ERBB2* (Her2/neu) exon 20 insertions and a few point mutations have been reported in 4% (34) of cases of lung adenocarcinoma in The Cancer Genome Atlas (TCGA) database and 3% of cases in the Lung Cancer Mutation Consortium project (35). In our experience with 145 cases of NSCLC that underwent NGS-based testing, 6.2% of cases showed exon 20 *ERBB2* alterations (36). One-third of these cases are also known to harbor *EGFR* mutations. Hence, incorporating next-generation sequencing is critical to effectively capture uncommon mutations and amplifications in *ERBB2* so that patients may be offered therapy directly targeted to their genomic alterations. Among the targeted agents available, tarloxotinib (NCT03805841), trastuzumab deruxtecan (NCT03505710), pyrotinib (NCT02500199), and poziotinib (NCT03318939) are just a few of the novel *ERBB2* inhibitors available in clinical trials. DESTINY Lung01 (37) is an ongoing multicenter trial investigating the role of trastuzumab deruxtecan in non-squamous NSCLC with activating *ERBB2* mutation/overexpression. The initial results show an ORR of 61.9% and a disease control rate (DCR) of 90.5% with a median PFS of 14 months.

## RNA-based alterations: fusions

### ALK

*ALK*-rearranged NSCLC has been reported in ∼7% of cases, and *EML4-ALK* was the first fusion identified in 2007 (38). These patients are usually young non-smokers with adenocarcinoma histology. Mutations and amplifications are also known to occur in the kinase domain of the *ALK* gene, which develop as resistance mechanisms to *ALK* TKIs. Crizotinib was the first *ALK* TKI approved for these cases, followed by the development of second- and third-generation TKIs (39).

Second-generation *ALK* inhibitors, ceritinib and alectinib, have now been both approved as first line treatments of *ALK*-rearranged NSCLC. The ALEX trial (40) demonstrated significantly improved PFS (34.8 months vs 10.9 months) and OS (NR vs 57.4 months; 5 years OS rate: 62.5% for alectinib vs 45.5% for crizotinib) with alectinib compared to crizotinib in treatment-naive *ALK*-positive NSCLC. Recent studies have addressed the impact of *ALK*-fusion variants, depending on breakpoints, on the response to *ALK* inhibitors. V1 and V3 *EML4-ALK* variants have been reported to be the most frequent. The traditionally used screening tool is the D5F3 monoclonal antibody by IHC, and trials like ALEX and PROFILE 1014 (41) have used the same for the detection of *ALK* rearrangement. Break-apart FISH was once considered confirmatory for the same; however, it suffers from signal intensity issues and observer-dependent variabilities. Owing to various breakpoints and fusion partners described, RT-PCR and Sanger sequencing are not optimal, owing to limited coverage and sensitivities. NGS using targeted RNA-based panels offers a one-stop solution not only to detect and characterize the fusion partners but also for response monitoring and the detection of additional resistance mutations (p.G1202R and p. L1196M) and other co-mutations like *TP53* (25), which are known to affect responses and prognosis. However, there are discordance instances reported; hence, IHC still forms the first step for detection.

### ROS1

Analogous to *ALK*, *ROS1* rearrangements have been described as drivers of NSCLC and have been reported in 1%–2% of cases (42). The patients are young and non-smokers, with a higher prevalence in Asians, similar to the profile of ALK-positive cases. Testing for *ROS1* has been recommended using break-part FISH, although IHC using the D4D6 rabbit monoclonal antibody has also been described with almost 100% sensitivity, which is optimal for screening. Similar to *ALK, ROS1* also has multiple fusion partners, which can be characterized using NGS, RT-PCR, and even NanoString technology. However, the combined sensitivity and specificity of IHC, followed by FISH, are higher than any of the other abovementioned technologies.

In two independent phase II prospective studies, the efficacy of crizotinib in *ROS1*-positive cases was evaluated, which depicted an ORR of 72% and 70%, respectively, with a median PFS of 19.2 months and 15.9 months (42). Analogous to *ALK*, resistance mutations are also described in the ROS1 kinase domain, namely, p. G2032R and p. D2033N, which are resistant to crizotinib but have shown some sensitivity to lorlatinib. In a single-arm phase 1–2 trial (NCT01970865) investigating the efficacy of lorlatinib in advanced NSCLC, 62% of patients who were TKI-naive showed an objective response. However, another substitution at 2032: p. G2032K has also shown lorlatinib resistance.

Co-mutations with *ROS1* are rare; however, performing NGS in this context is relevant, owing to solvent front resistance mutations, which develop during the course of the disease.

### RET

*RET* fusions have been reported in 1%–2% of cases of NSCLC (10). The recent approval of selpercatinib, following the results from LIBRETTO-001 (43) for *RET*-rearranged NSCLC cases, has sparked keen interest in the detailed characterization of clinicopathologic features and response outcomes of this disease. The current practice of performing single-gene testing does not incorporate *RET* fusion detection, and hence, NGS-based panel testing may prove promising.

Testing for *RET* traditionally has been using break-apart FISH and was not recommended upfront. With newer drugs making their way into the clinic, it is important to test the same upfront. Panel-based NGS testing using RNA-based panels like the Oncomine fusion panel, anchored multiplex PCR, Illumina TruSight, and the FDA-approved FoundationOne incorporate *RET* gene alterations. Recently, resistance mutation *RET* p. S904F has been identified, which is resistant to vandetanib. Hence, NGS with both RNA and DNA are now mandatory in the first line.

### NTRK

NTRK fusions are detected across multiple pediatric and adult malignancies. The frequency of these fusions varies from <1% in malignancies like lung, colorectal, pancreatic, breast cancers, melanoma, and other solid or hematological cancers (44). They have gained importance as tissue-agnostic markers, owing to the development of specific inhibitors like entrectinib and larotrectinib, following the results from trials like START-TRK. In February 2015, entrectinib was granted FDA Orphan Drug Designation for *NTRK*-positive NSCLC and colorectal neoplasms. Larotrectinib activity has been evaluated in three trials: a phase I trial in adults (NCT02122913), a phase I/II trial in pediatric patients (SCOUT, NCT02637687), and a phase II trial involving adults and adolescents (NAVIGATE, NCT02576431) (45).

The current ESMO recommendations (46) incorporate the use of IHC for *NTRK* testing both for screening in the case of tissue unavailability in common cancers and confirmatory in *NTRK*-enriched tumors, after NGS-based testing. IHC, although less sensitive and variable for each of the three *NTRK1*, 2, and 3 with variable staining patterns, is still appealing, owing to its low cost and good sensitivity, making it a good screening tool.

## Emerging biomarker: NRG fusion

*NRG1* fusions have emerged as uncommon biomarkers, especially in invasive mucinous adenocarcinoma (47). They activate *Her2/Her3* signaling, and anecdotal case reports have shown durable responses to afatinib (48). However, this has yet to be validated in controlled trials. Hence, owing to the paucity of the literature on this gene, it is not yet included in upfront diagnostic tumor profiling, and no NGS panels currently incorporate it. However, future RNA sequencing and fusion panels will have to eventually include it.

**FIGURE 2:**
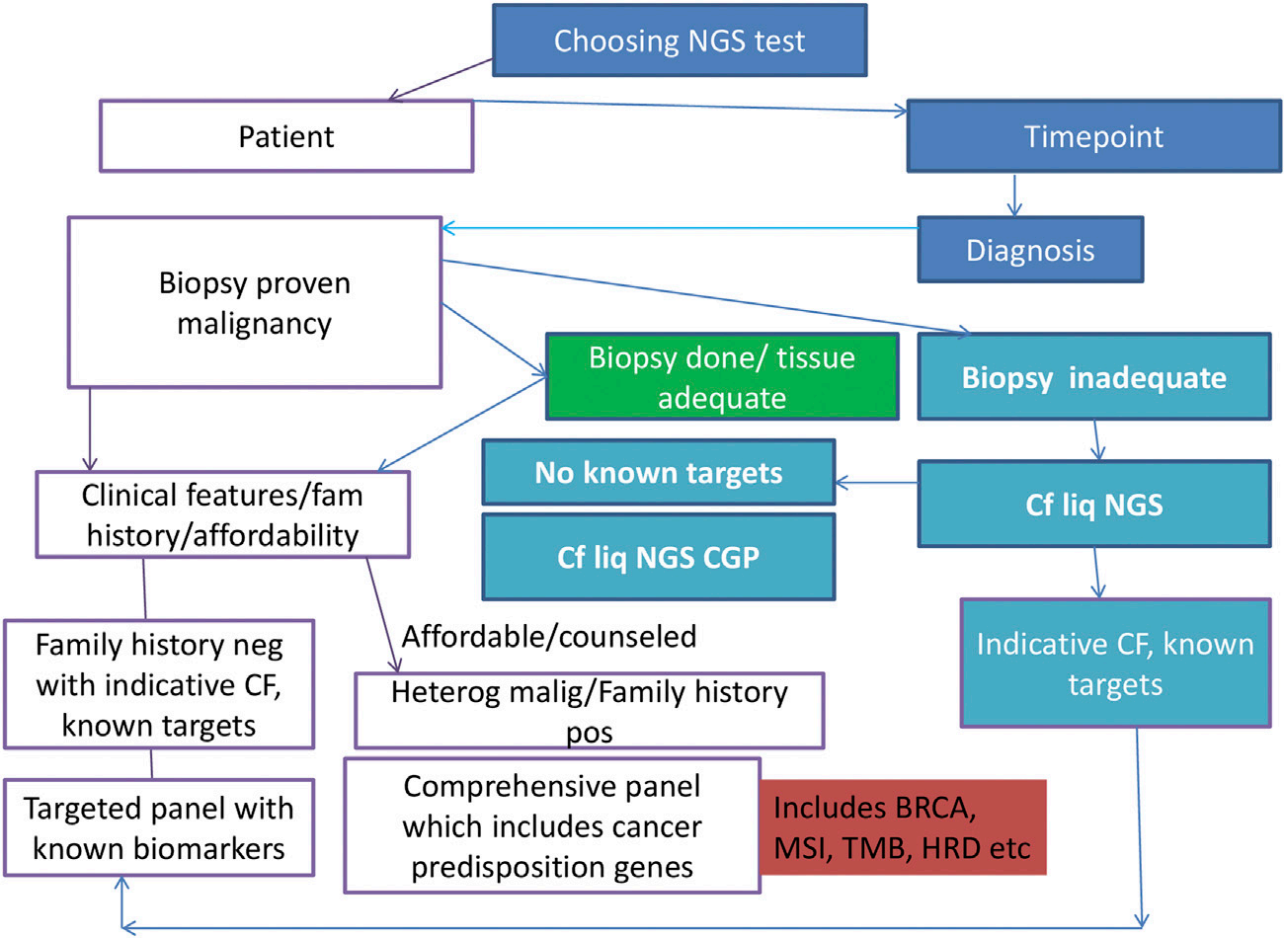
Algorithm/flow diagram depicting the best-proposed panel for molecular profiling in lung carcinoma. NSCLC, non-small cell lung carcinoma; DNA, deoxyribonucleic acid; RNA, ribonucleic acid; NGS: next-generation sequencing.

The proposed panel of genes and biomarkers to be tested in NSCLC is depicted in Figure 2. The ongoing clinical trials for the biomarkers are detailed in Table 3.

**TABLE 3:** Current clinical trials with trial IDs for biomarkers in lung carcinoma.

Biomarker	• Trial	• Trial number	• Drug(s)
*EGFR*	• Phase I • Phase III • Phase 1	• NCT02609776 • NCT04181060 • NCT03755102	• Amivantamab • Osimertinib • Dacomitinib
*ALK*	• Phase II	• NCT02927340	• Lorlatinib
*ROS1*	• Phase II	• NCT02927340	• Lorlatinib
*MET*	• Geometry Mono 1 (Phase II)	• NCT02414139	• Capmatinib
*BRAF*	• Phase II	• NCT03915951	• Encorafenib + Binimetinib
*KRAS*	• CodeBreak 100 (phase II) • Krystal-1	• NCT03600883 • NCT03785249	• AMG-510 (sotorasib) • MRTX849 (adagrasib)
*RET*	• A LUNG-MAP Treatment trial (phase II) • Phase I/2	• NCT04268550 • NCT03037385	• Selpercatinib • Pralsetinib
*NTRK*	• RXDX 101 (phase II)	• NCT02568267	• Entrectinib
*ERBB2*	• Phase II • Phase II basket trial	• NCT03318939 • NCT01953926	• Poziotinib • Neratinib

## Conclusions and key points

Targeted therapies and precision medicine have paved the way for broader molecular testing, offering an insight into disease biology and evolution. However, clinicians need to be aware that sequential single-gene testing results in tissue exhaustion with higher false negative rates. Additionally, whole exome/whole genome approaches are not suitable for all samples. Targeted NGS panels may prove to be more promising than sequential single-gene testing. Test performances may vary due to differences in sensitivities, specificities, depth, and coverage, and hence, the test performance should be a part of the final molecular report rendered to the clinician in order to deliver appropriate therapy. Clinical interpretation should be made with utmost care and involve tumor board discussions, prior to the initiation of therapy.

From the data presented on various biomarkers in NSCLC, it is clearly evident that NGS-based testing forms the foundation in this constantly evolving field of precision medicine. Testing strategies must advance to take into account the ever-expanding list of new biomarkers, new drugs, and the need to not only diagnose but also to monitor disease responses.
